# Nuclear Translocation of *Acinetobacter baumannii* Transposase Induces DNA Methylation of CpG Regions in the Promoters of *E-cadherin* Gene

**DOI:** 10.1371/journal.pone.0038974

**Published:** 2012-06-07

**Authors:** Dong Chan Moon, Chul Hee Choi, Su Man Lee, Jung Hwa Lee, Seung Il Kim, Dong Sun Kim, Je Chul Lee

**Affiliations:** 1 Department of Microbiology, Kyungpook National University School of Medicine, Daegu, Korea; 2 Department of Periodontology, University of Florida, Gainesville, Florida, United States of America; 3 Department of Anatomy, Kyungpook National University School of Medicine, Daegu, Korea; 4 Division of Life Science, Korea Basic Science Institute, Daejeon, Korea; University of Padova, Italy

## Abstract

Nuclear targeting of bacterial proteins has emerged as a pathogenic mechanism whereby bacterial proteins induce host cell pathology. In this study, we examined nuclear targeting of *Acinetobacter baumannii* transposase (Tnp) and subsequent epigenetic changes in host cells. Tnp of *A. baumannii* ATCC 17978 possesses nuclear localization signals (NLSs), _225_RKRKRK_230_. Transient expression of *A. baumannii* Tnp fused with green fluorescent protein (GFP) resulted in the nuclear localization of these proteins in COS-7 cells, whereas the truncated Tnp without NLSs fused with GFP were exclusively localized in the cytoplasm. *A. baumannii* Tnp was found in outer membrane vesicles, which delivered this protein to the nucleus of host cells. Nuclear expression of *A. baumannii* Tnp fused with GFP in A549 cells induced DNA methylation of CpG regions in the promoters of *E-cadherin* (*CDH1*) gene, whereas the cytoplasmic localization of the truncated Tnp without NLSs fused with GFP did not induce DNA methylation. DNA methylation in the promoters of *E-cadherin* gene induced by nuclear targeting of *A. baumannii* Tnp resulted in down-regulation of gene expression. In conclusion, our data show that nuclear traffic of *A. baumannii* Tnp induces DNA methylation of CpG regions in the promoters of *E-cadherin* gene, which subsequently down-regulates gene expression. This study provides a new insight into the epigenetic control of host genes by bacterial proteins.

## Introduction

*Acinetobacter baumannii* is an important opportunistic pathogen that causes a variety of human infections in both community and hospitals [1], [2]. *A. baumannii* infection causes a high mortality rate in patients with mechanical ventilation and a fatal underlying disease [3]. The fatality of patients infected with *A. baumannii* is primarily due to host factors, but bacterial virulence factors such as biofilm formation [4], [5], serum resistance [6], [7], bacterial adherence to host cells [8], and host cell death [9], [10] are also associated with pathogenic processes and disease development.

Nuclear targeting of bacterial proteins has emerged as a pathogenic mechanism whereby bacterial proteins can directly interact with nuclear molecules or indirectly disturb signal transduction pathways, which result in host cell pathology [11]. To date, very few bacterial proteins, including cytolethal distending toxins of Gram-negative bacteria [12]–[14], IpaH9.8 and OspF of *Shigella* species [15], [16], SspH1 of *Salmonella enterica* [17], YopM of *Yersinia* species [18], and a novel nuclear effector (NUE) of *Chlamydia trachomatis* [19], and outer membrane protein A of *A. baumannii* [9], have been found to target the nuclei of host cells and induce cell pathology. However, whole genome analysis revealed that *A. baumannii*, *Escherichia coli*, *Helicobacter pylori*, *Pseudomonas aeruginosa*, and *Shigella sonnei* were found to carry several proteins with nuclear localization signals (NLSs) [20], [21]. NLSs are recognized by nuclear transport proteins, importins, and a complex of the NLS-carrying proteins and importins is transported to the nucleus through the nuclear pore complex (NPC) [22], [23]. These results suggest that pathogenic bacteria may employ a strategy to target their effector proteins to the nuclei of host cells.

Epigenetic alterations are heritable and reversible changes that alter gene expression without changing the primary DNA sequence and comprise DNA methylation, histone modification, and small, noncoding RNAs [24]. They are involved in transcriptional changes and decisive events determine cell fate and phenotype. DNA methylation occurs on C5 of the cytosine in the dinucleotide CpG sites and closely interacts with histone modifications. In addition, it is required for chromosomal stability, and is a powerful mechanism for maintaining the suppression of gene activity. Accumulation evidence indicates that alteration of DNA methylation directly or indirectly contributes to the susceptibility and development of many complex or multifactorial disease [25]. Bacterial infection has recently been shown to induce aberrant DNA methylation of CpG regions in the promoters of host genes, which allow a pathogen to inhibit transcription of host genes. *Campylobacter rectus* induces hypermethylation in the promoter region of the *Igf2* gene [26]. *H. pylori* infection induces CpG methylation in the promoter regions of mismatch repair and tumor suppressor genes, which are associated with the initiation and progression of gastric cancer [27]–[29]. In addition to chronic bacterial infection, uropathogenic *E. coli* also induces DNA methylation in *CDKN2A* (*p16^INK4A^*) and results in epigenetic down-regulation of this gene in uroepithelial cells [30]. Induction of aberrant DNA methylation and subsequent down-regulation of host genes by bacterial infection are considered to be a new pathogenic mechanism of bacteria.

We previously predicted the NLS-carrying proteins among the open reading frames (ORFs) of *A. baumannii* ATCC 17978 based on NLS sequences and found that *A. baumannii* transposase (Tnp) possessed NLSs, RKRKRK, between amino acid positions 225 and 230 [31]. To obtain a better understanding of *A. baumannii* pathogenesis regarding nuclear targeting of bacterial proteins, we examined secretion of *A. baumannii* Tnp from bacteria and its delivery to host cells, nuclear targeting of *A. baumannii* Tnp, and epigenetic changes and gene expression of host cells. We report here that *A. baumannii* Tnp induces DNA methylation in CpG regions of *E-cadherin* (*CDH1*) gene via nuclear targeting, which subsequently down-regulates expression of this gene.

## Results

### Nuclear targeting of *A. baumannii* Tnp via NLS sequences

**Figure 1:**
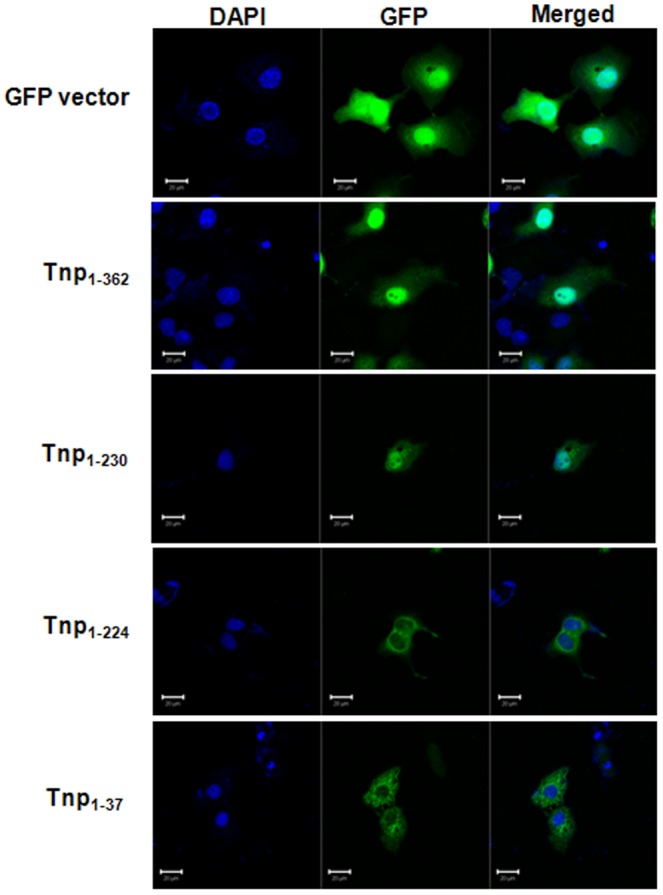
*A. baumannii* transposase targets in the nucleus of host cells via NLSs. COS-7 cells were transfected with the plasmid constructs of transposase gene cloned in the destination vector pcDNATM6.2/N-EmGFP-DEST and incubated for 24 h. The subcellular localization of transposase proteins fused with GFP was observed by confocal laser microscopy. Two *A. baumannii* transposase proteins with NLSs, Tnp_1–362_ and Tnp_1–230_, were located in the nuclei of host cells, whereas transposase proteins without NLSs, Tnp_1–37_ and Tnp_1–224_, were located in the cytoplasm.

Tnp of *A. baumannii* ATCC 17978 (NCBI accession no. gi|126640304) was composed of 362 amino acids and was predicted to carry the putative NLSs, _225_RKRKRK_230_ [31]. To determine whether *A. baumannii* Tnp targeted the nuclei of host cells, the full-length *A. baumannii* Tnp gene was cloned into pcDNA™6.2/N-EmGFP-DEST using the Gateway recombinational cloning system (Invitrogen) and the constructed plasmids were transfected into COS-7 cells. As a control, COS-7 cells were transfected with the destination vector pcDNA™6.2/N-EmGFP-DEST. The green fluorescent protein (GFP) composed of a molecular mass of 27 kDa, was observed in both the cytoplasm and the nucleus of COS-7 cells transfected with the destination vector. GFP behaves within the exclusion limit of NPC and passively diffuses into the nuclei of host cells, whereas GFP-tagged *A. baumannii* Tnp fusion proteins composed of a molecular mass of 66.8 kDa, are exclusively present in the nuclei (Fig. 1). To determine whether nuclear targeting of *A. baumannii* Tnp was dependent on NLSs, three mutant clones, Tnp_1–37_, Tnp_1–224_, and Tnp_1–230_, fused with GFP were constructed and their subcellular localization was determined by confocal laser microscopy. Two *A. baumannii* Tnp mutant clones without NLSs, Tnp_1–37_ and Tnp_1–224_, were present in the cytoplasm of host cells, whereas the mutant clone with NLSs, Tnp_1–230_, appeared in the nuclei (Fig. 1). These results suggest that *A. baumannii* Tnp targets the nuclei of host cells via functional NLSs.

### Delivery of *A. baumannii* Tnp to host cells via outer membrane vesicles (OMVs)

**Figure 2:**
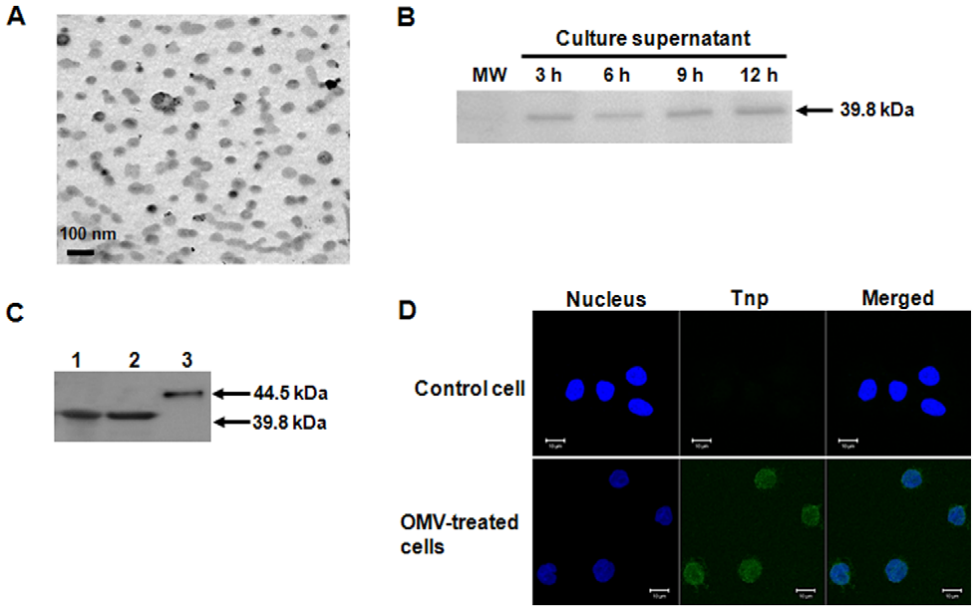
*A. baumannii* OMVs deliver transposase to the nucleus of host cells. (A) TEM observation of OMVs from *A. baumannii* ATCC 17978. (B) Detection of *A. baumannii* transposase in the bacterial culture supernatant. Bacteria were cultured in LB broth and proteins in the culture supernatants were subjected to 12% SDS-PAGE and Western blot analysis using the polyclonal anti-mouse transposase antibody. (C) Secretion of *A. baumannii* transposase from bacteria via OMVs. Bacterial cell lysates (lane 1), OMVs (lane 2), and recombinant *A. baumannii* transposase (lane 3) were subjected to 12% SDS-PAGE and Western blot analysis using the polyclonal anti-mouse transposase antibody. (D) COS-7 cells were treated with *A. baumannii* OMVs (20 µg/ml of protein concentrations) for 24 h. Cells were fixed, permeabilized with Triton X-100, and stained with a mouse anti-*A. baumannii* transposase polyclonal immune sera, followed by Alexa Fluor 488-conjugated mouse immunoglobulin G (green). DAPI was used to stain the nuclei (blue). Subcellular distribution of *A. baumannii* transposase was analyzed by confocal microscopy. Analytical sectioning was performed from the top to the bottom of the cells. The figure represents all projections of the sections in one picture.

Translocation to the cytoplasm of host cells is an essential step for nuclear targeting of bacterial proteins. We previously demonstrated that *A. baumannii* ATCC 19606^T^ and a clinical isolate DU202 secreted OMVs [32], [33]. Since OMVs derived from *A. baumannii* and *E. coli* contained many bacterial proteins that originated from the outer membrane, periplasmic space, inner membrane, and cytoplasm [32]–[34], we determined whether *A. baumannii* Tnp was secreted from bacteria via OMVs. *A. baumannii* ATCC 17978 was cultured in Luria-Bertani (LB) broth and OMVs were purified from the culture supernatants. Transmission electron microscopic (TEM) analysis showed that *A. baumannii* ATCC 17978 secreted OMVs during *in vitro* culture (Fig. 2A). To verify the presence of OMVs, bacterial lysates, culture supernatants, and OMVs were separated by 12% sodium dodecyl sulfate-polyacrylamide gel electrophoresis (SDS-PAGE). Protein profiles were different between three samples (data not shown), suggesting that OMVs purified from *A. baumannii* ATCC 17978 were not bacterial lysates or artifacts. To determine whether *A. baumannii* ATCC 17978 could secrete Tnp *in vitro* culture, bacterial culture supernatants were subjected to Western blot analysis using polyclonal anti-mouse *A. baumannii* Tnp antibody. *A. baumannii* Tnp, which has a molecular mass of 39.8 kDa, was detected in the bacterial culture supernatant (Fig. 2B). To verify the presence of Tnp in the *A. baumannii* OMVs, bacterial cell lysates and OMVs were subjected to Western blot analysis. As a control, recombinant *A. baumannii* Tnp, which has a molecular mass of 44.5 kDa, was used. *A. baumannii* Tnp with a molecular mass of 39.8 kDa was detected in both the bacterial cell lysates and OMVs (Fig. 2C). Next, to determine whether OMVs could deliver *A. baumannii* Tnp to host cells, COS-7 cells were treated with *A. baumannii* OMVs and the subcellular distribution of *A. baumannii* Tnp was analyzed by confocal laser microscopy. *A. baumannii* Tnp was mainly distributed in the nuclei of COS-7 cells (Fig. 2D). These results suggest that *A. baumannii* OMVs deliver their component Tnp to the cytoplasm of host cells.

### DNA methylation of CpG regions in the promoters of *E-cadherin* gene and down-regulation of gene expression by nuclear targeting of *A. baumannii* Tnp

**Figure 3:**
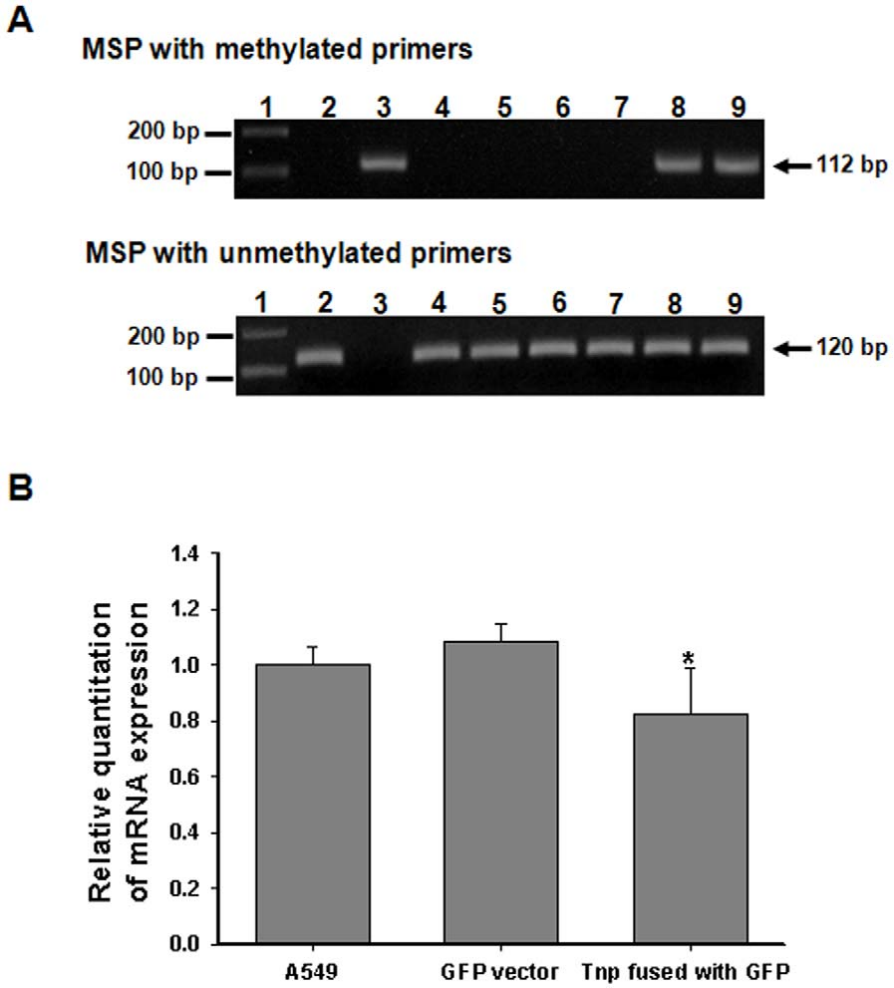
Nuclear targeting of *A. baumannii* transposase specifically induces DNA methylation in CpG regions and down-regulates expression of *E-cadherin* gene. (A) A549 cells were transfected with *A. baumannii* transposase clones and incubated for 48 h. The genomic DNA was purified and methylation-specific PCR with methylated and unmethylated primers was performed as described in materials and methods. Lane 1, molecular size marker; 2, unmethylated DNA; 3, methylated DNA; 4, A549 cells; 5, A549 cells transfected with the destination vector pcDNA™6.2/N-EmGFP-DEST; 6, A549 cells transfected with plasmid constructs of Tnp_1–37_; 7, A549 cells transfected with plasmid constructs of Tnp_1–224_; 8, A549 cells transfected with plasmid constructs of Tnp_1–230_; 9, A549 cells transfected with plasmid constructs of Tnp_1–362_. (B) A549 cells were transfected with *A. baumannii* transposase clones and incubated for 48 h. Total RNA was extracted and qRT-PCR was performed as described in materials and methods. Data are presented as mean ± SD of triplicate determinations. Asterisks indicate a statistically significant difference between A549 cells transfected with the empty destination vector and plasmid constructs of *A. baumannii* transposase fused with GFP (student's t-test *p*<0.05).

To determine whether nuclear targeting of *A. baumannii* Tnp induced cellular damage, cells were transfected with plasmid constructs containing the full-length *A. baumannii* Tnp gene cloned in the pcDNA™6.2/N-EmGFP-DEST and incubated for 48 h. The viability of COS-7 cells transfected with the full-length *A. baumannii* Tnp gene was slightly increased (126±2.8%) as compared to that of COS-7 cells transfected with the empty destination vector. Expression of *A. baumannii* Tnp fused with GFP in the nuclei of A549 cells did not induce any morphological change relative to control cells transfected with the destination vector (Fig. 1). To determine whether *A. baumannii* Tnp induced epigenetic changes in host cells, A549 cells were transfected with plasmid constructs of the full-length *A. baumannii* Tnp gene cloned in pcDNA™6.2/N-EmGFP-DEST and incubated for 48 h. A549 cells that originated from human lung carcinoma were used because the respiratory tract is the most common infection site of *A. baumannii* [2]. Genomic DNA was extracted from A549 cells and methylation-specific polymerase chain reaction (MSP) was performed using primers specific for the CpG regions of *p16^INK4A^*, *hMLH1*, and *E-cadherin* genes, which are involved in inhibiting cell cycle progression, DNA mismatch repair, and adhesion of epithelial cells to one another, respectively [35]–[39]. *A. baumannii* Tnp specifically induced DNA methylation of CpG regions in the promoters of *E-cadherin* gene (Fig. 3A), but not in CpG regions of *p16^INK4A^* and *hMLH1* (data not shown). To determine whether DNA methylation of CpG regions in the promoter of *E-cadherin* gene was dependent on nuclear targeting of *A. baumannii* Tnp, A549 cells were transfected with three mutant clones, Tnp_1–37_, Tnp_1–224_, and Tnp_1–230_, fused with GFP and then MSP specific for the CpG regions of *E-cadherin* gene was performed. The truncated Tnp_1–230_ with NLSs induced DNA methylation, whereas the two mutant clones without NLSs, Tnp_1–37_ and Tnp_1–224_, did not induce DNA methylation (Fig. 3A). An aberrant DNA methylation in the promoters of genes can down-regulate transcription level. We determined mRNA expression of *E-cadherin* gene in A549 cells transfected with plasmid constructs of the full-length of *A. baumannii* Tnp fused with GFP. When transfection efficiency reached to 60–70%, total RNA of cells was harvested and quantitative reverse transcriptase-PCR (qRT-PCR) was performed. As a control, A549 cells were transfected with pcDNA™6.2/N-EmGFP-DEST vector. *A. baumannii* Tnp down-regulated mRNA expression of *E-cadherin* gene (0.82±0.16) as compared to the empty destination vector (1.0±0.06) (*p*<0.05) (Fig. 3B). These results suggest that nuclear targeting of *A. baumannii* Tnp specifically induces DNA methylation of CpG regions in the promoters of *E-cadherin* gene and then down-regulates gene expression.

## Discussion

Bacterial proteins that target the nucleus of host cells play a crucial role in bacterial pathogenesis. In this study, we demonstrated that *A. baumannii* Tnp is a new bacterial effector that induces DNA methylation of CpG regions in the promoters of *E-cadherin* gene via nuclear targeting. *A. baumannii* Tnp does not only catalyze ‘cut-and-paste’ reactions, which promotes the movement of DNA segments to new sites, but also induces epigenetic modification of host genes. This is the first study to report that the *A. baumannii* protein directly induces epigenetic alteration via nuclear targeting.

Whole genome analysis of bacteria is a highly useful tool to predict nuclear targeting proteins based on NLSs. We identified 34 proteins with the putative NLSs among the 3,367 ORFs of *A. baumannii* ATCC 17978 [31]. Of the *A. baumannii* proteins predicted to carry the putative NLSs, 14 were found to target in the nuclei of host cells. Among the nuclear targeting proteins identified, we selected *A. baumannii* Tnp to determine the DNA methylation of CpG regions in the promoters of genes because several bacterial transposons encoded their own DNA modifying enzymes to regulate gene expression [40]. Aberrant DNA methylation of CpG regions in the promoters of host genes allows a pathogen to inhibit or down-regulate transcription of specific genes, which may alter host cell biology. DNA methylation in CpG regions of host genes by bacterial infection has been demonstrated in several previous studies [26], [29], [30]. Bacterial infection and inflammatory mediators released from host cells have been found to trigger CpG methylation in the promoters of eukaryotic genes [41]. However, specific bacterial molecules that induce DNA methylation in promoters of host genes have not yet been identified.

Nuclear targeting of *A. baumannii* Tnp did not induce cytotoxicity of host cells, although several nuclear targeting proteins of *A. baumannii*, such as transcriptional regulator, 50S ribosomal protein L20, putative transcriptional regulator, and DNA cytosine methyltransferase, induce host cell death [31]. Instead, *A. baumannii* Tnp specifically induced DNA methylation in the CpG regions of *E-cadherin* gene, but not in the CpG regions of *p16^INK4A^* and *hMLH1*. Aberrant DNA methylation in CpG regions of *E-cadherin* gene was specifically induced by nuclear targeting of *A. baumannii* Tnp with NLSs, but not induced by cytoplasmic localization of mutant *A. baumannii* Tnp without NLSs. Our results suggest that *A. baumannii* Tnp may exert effects on epigenetic alterations of host cells after nuclear targeting. Moreover, we demonstrated that DNA methylation in the CpG regions of *E-cadherin* gene down-regulates expression of this gene. Expression of *E-cadherin* gene was significantly different between A549 cells transfected with the empty destination vector and plasmid clones of *A. baumannii* Tnp fused with GFP. We did not determine the molecular mechanisms of DNA methylation in the CpG regions of *E-cadherin* gene such as activation of DNA methyltransferases, but this study identified a novel pathogenic mechanism by which bacterial proteins regulate expression of host genes via epigenetic alterations.

There are some variations in methylation frequency of tumor-associated genes in tumors. We used A549 cells originated from human lung carcinoma for DNA methylation of CpG regions in the promoters of tumor-associated genes because the respiratory tract is the most common site for colonization and infection of *A. baumannii*. *E-cadherin* and *p16^INK4A^* genes are frequently methyated in lung cancer, whereas *hMLH1* gene is rarely methylated and its methylation correlates with late stage of lung cancer [42]. It is thus plausible to guess that acute and transient transfection of *A. baumannii* Tnp cannot induce DNA methylation of *hMLH1* gene in A549 cells. E-cadherin is the key components for adherence junctions between epithelial cells, which allow the body to maintain internal homeostasis as a physical barrier [43]. Many pathogenic bacteria can destroy junctional complexes that comprise the protective functions of epithelial cells. In particular, the virulence factors CagA and VacA secreted by *H. pylori* disrupt the tight and adherent junctions and cytoskeleton architecture, and increase cell proliferation through gene modification, finally contributing to gastric carcinogenesis [44]. Interestingly, E-cadherin also has a growth suppressor function by inducing cell cycle arrest via up-regulation of the cyclin-dependent kinases, p27 [45]. It is thus tempting to speculate that *E-cadherin* gene may be a good target of *A. baumannii* Tnp.

We found that *A. baumannii* Tnp was secreted from bacteria and delivered to host cells via OMVs. To determine the mechanisms underlying secretion and delivery of *A. baumannii* Tnp to host cells, recognition sites necessary for type I or II secretion systems were searched. However, *A. baumannii* Tnp did not harbor signal peptides or recognition sites necessary for type I or II secretion systems. *A. baumannii* does not have type III or IV secretion systems, although several genes encoding type IV secretion systems have been found [46]. Instead, *A. baumannii* OMVs contained more than 100 proteins derived from the outer membrane, periplasmic space, inner membrane, and even cytoplasm [32], [33]. *A. baumannii* Tnp was not found in the proteome of OMVs from *A. baumannii* ATCC 19606^T^ and DU202 [32], [33], but this nuclear targeting protein was identified in OMVs from *A. baumannii* ATCC 17978 using Western blot analysis in this study. Discrepancy of *A. baumannii* Tnp in the OMVs is possibly due to limitations in the proteomic analysis or differences between *A. baumannii* strains.

In conclusion, the present study demonstrated that nuclear targeting of *A. baumannii* Tnp induces DNA methylation of CpG regions in the promoters of *E-cadherin* gene and down-regulates expression of this gene. Our study may contribute to a novel pathogenic mechanism by which bacterial proteins directly regulate gene expression of host cells via epigenetic alterations.

## Materials and Methods

### Bacterial strains and DNA manipulations

*A. baumannii* ATCC 17978 was grown on blood agar plates at 37°C. Genomic DNA was purified from bacteria cultured in LB broth using a genomic DNA preparation kit (SolGent, Korea) and then used as a template for PCR. *E. coli* DH5α and BL21 (DE3) were used for DNA cloning and production of recombinant proteins, respectively. *E. coli* strains were grown on blood agar plates or in LB broth at 37°C. Routine DNA manipulations were performed as previously described [47] or according to the manufacturer's instructions of the reagents used.

### Cell culture

Two eukaryotic cell lines, COS-7 originating from African green monkey kidney and A549 cells originating from human lung carcinoma, were purchased from Korean Cell Line Bak (Seoul, Korea) and used in this study. COS-7 cells were grown in Dulbecco's modified Eagle's medium (HyClone) supplemented with 10% fetal bovine serum (FBS; HyClone), 2.0 mM _L_-glutamine, 100 U/ml penicillin, and 20 µg/ml streptomycin at 37°C in 5% CO_2_. A549 cells were grown in RPMI 1640 medium (HyClone) supplemented with 10% FBS, 2.0 mM _L_-glutamine, 100 U/ml penicillin, and 20 µg/ml streptomycin at 37°C in 5% CO_2_. The cultured cells were seeded in 6-well or 12-well tissue culture plates to purify genomic DNA or transfect plasmid constructs, respectively.

### Construction and expression of *A. baumannii* Tnp-GFP fusion proteins

Genomic DNA was purified from *A. baumannii* ATCC 17978 and used as a template to perform PCR. The Gateway recombinational cloning system was used for these experiments. The specific primer set used for the full-length *A. baumannii* Tnp was as follows: forward primer-5′-AAA AAG CAG GCT CCA CCA TGA TCG TAG GGT ATT ACC TAT C-3′, reverse primer-5′-AGA AAG CTG GGT TCC TTA AAT CGT CAA ATG CAG TTA A-3′. To generate *A. baumannii* Tnp mutant clones, a forward primer (5′-GGG GAC AAG TTT GTA CAA AAA AGC AGG CTC CAC CAT GAT CGT AGG GTA TTA CCT ATC-3′) and three reverse primers (5′-GGG GAC CAC TTT GTA CAA GAA AGC TGG GTT AAT ATC GAG TTC GAT CAA CTT ACG-3′ for Tnp_1–37_, 5′-GGG GAC CAC TTT GTA CAA GAA AGC TGG GTT ATT ATT GTC CTC TGG ATC AAT AG-3′ for Tnp_1–224_, and 5′-ACA AGA AAG CTG GGT TTT TTC TTT TGC GCT TTC GAT TAT T-3′ for Tnp_1–230_) were used. PCR was performed in a total volume of 20 µl containing the following: 1.5 U Platinum *Pfx* DNA polymerase (Invitrogen, USA), 2 µl of 10× *Pfx* amplification buffer, 0.3 mM dNTP mixture, 1 mM MgSO_4_, 0.3 µM of each primer, and template DNA (100 ng). The PCR products were amplified again with the *att*B adapter primers (5′-GGG GAC AAG TTT GTA CAA AAA AGC AGG CTC CAC C-3′ and 5′-GGG GAC CAC TTT GTA CAA GAA AGC TGG GTT-3′), which generated the full-length *att*B1 and *att*B2 sites flanking *A. baumannii* Tnp ORFs. The Gateway-compatible amplified gene was recombined into the pDONR221 vector (Invitrogen) using the BP reactions. The plasmid pDONR207 was mixed with 2 µl of the *att*B-linked PCR product in 15 µl of BP reaction mixture containing 3 µl BP Clonase I enzyme mix (Invitrogen). After incubation at 25°C for 60 min, proteinase K (4 µg in 1.5 µl) was added and then each reaction was incubated at 37°C for 10 min. BP reaction mixtures were used directly for bacterial transformation. Aliquots (5 µl) of the entry clone were used to transform *E. coli* DH5α cells (Library Efficiency) and bacteria were plated on LB medium containing 50 µg/ml of gentamicin. A single colony from the transformed plates was tested by colony-PCR with specific primers for the *A. baumannii* Tnp gene and sequenced using an ABI Prism 3730XL Analyzer (Applied Biosystems). The entry clone was used for the generation of GFP-tagged clones or production of recombinant proteins in a reaction mixture containing 2 µl LR Clonase II enzyme mix (Invitrogen), 150 ng pcDNA™6.2/N-EmGFP-DEST vector (Invitrogen) for the GFP-tagged clones or pET160-DEST (Invitrogen) for the production of recombinant proteins, and TE buffer (pH 8.0). After incubation at 25°C for 3 h, proteinase K (1 µg/µl) was added and each reaction was further incubated at 37°C for 10 min. The LR reactions were used to transform *E. coli* DH5α or BL21 (DE3).

### Transfection of the constructed plasmids in host cells

The plasmid constructs obtained from the LR reactions were purified using an Exprep™ plasmid SV kit (GeneAll, Korea) and plasmid DNA was diluted in Opti-MEM MEM I medium (Invitrogen). The diluted DNA (1.6 µg) was reacted with 4 µl of Lipofectamine™ 2000 (Invitrogen) that had been diluted in Opti-MEM MEM I medium for 45 min at room temperature. The mixture was added to 1×10^5^ cells and the cells were incubated in a CO_2_ incubator for 24 h. The subcellular localization of GFP-tagged proteins was observed using a confocal laser microscope (Carl Zeiss).

### Production of recombinant *A. baumannii* Tnp proteins

The plasmid constructs obtained from the LR reactions using the Gateway cloning system were transformed into *E. coli* BL21 (DE3) and recombinant proteins were overexpressed after induction with 1 mM of isopropyl β-D-1-thiogalactopyranoside at 37°C for 4 h. Recombinant proteins were purified using a nickel-column (Amersham Biosciences) and endotoxins were removed by polymyxin B-coated beads (Sigma). The protein concentration was determined using a modified BCA assay (Thermo Scientific). Concentrations of endotoxins were determined using a *Limulus* Amebocyte lysate test kit (Sigma) and the quantity of endotoxin in the recombinant proteins was ≤0.01 ng/mg.

### Purification of OMVs

OMVs were prepared from *A. baumannii* ATCC 17978 as previously described [34], [48]. Bacteria were grown in LB broth at 37°C with shaking until the optical density at 600 nm reached 1.0. After removing bacterial cells, culture supernatants were filtered through a 0.2 µm hollow fiber membrane equipped with QuixStand Benchtop System (GE Healthcare) to remove residual bacterial debris. The samples were then concentrated via ultrafiltration with a QuixStand Benchtop System using a 500 kDa hollow fiber membrane (GE Healthcare). The OMV fractions were ultracentrifugated at 150,000× g at 4°C for 3 h. The purified OMVs were resuspended in phosphate-buffered saline (PBS) and checked for sterility. The OMVs were applied to copper grids and stained with 2% uranyl acetate. The OMVs were visualized using a transmission electron microscope (Hitachi, Japan) that was operated at 120 kV.

### Western blot analysis

Bacteria were cultured in LB broth for the indicated time periods and then culture supernatants were collected. Proteins in the culture supernatants were precipitated with trichloroacetic acid. Protein concentrations of each bacterial culture supernatant and bacterial cell lysates were quantified using a modified BCA assay (Thermo Scientific). The samples were separated by 12% SDS-PAGE, followed by electrotransfer onto nitrocellulose membranes (Hybond-ECL; Amersham Pharmacia Biotech). Membrane blots were blocked in 5% non-fat skim milk and incubated with a mouse anti-*A. baumannii* Tnp immune sera, which were produced in our laboratory. The membranes were incubated with a secondary antibody coupled to horseradish peroxidase and developed using an enhanced chemiluminescence system (Amersham Pharmacia Biotech).

### Cytotoxicity assay

The cellular cytotoxicity was measured using the Premix WST1 cell proliferation assay system (TaKaRa) [10]. Cells were transfected with the destination vector pcDNA™6.2/N-EmGFP-DEST and *A. baumannii* Tnp constructs cloned in the destination vector, and then incubated for 48 h. When transfection efficiency of the cloned plasmids reached to 65–70%, WST1 assay was performed. Cellular growth was measured at 450 nm for 3 h after treatment with WST1.

### Methylation-specific PCR

A549 cells were transfected with plasmid constructs of *A. baumannii* Tnp cloned in the pcDNA™6.2/N-EmGFP-DEST vector and the empty destination vector. After 48 h of incubation, genomic DNA was purified using SolGent™ Genomic DNA prep kit (SolGent, Korea). The methylation status of the target genes was determined using a methylation-specific PCR with primers specific for the methylated and unmethylated alleles of each gene after treating the genomic DNA with sodium bisulfite [49]–[51]. The primer sequences, annealing temperatures, and the expected sizes of PCR products are summarized in Table 1. Briefly, 1 µg of DNA was denatured with sodium hydroxide and modified with sodium bisulfite and DNA samples were purified using Wizard DNA purification resin (Promega). The sample DNA was treated with sodium hydroxide again, precipitated with ethanol, and resuspended in distilled water. All PCR amplification steps were carried out using reagents supplied in a GeneAmp DNA Amplification Kit with AmpliTaq Gold (PE Applied Biosystems) on PTC-100 (MJ Research). The CpGenome™ universal methylated and unmethylated DNA was used as a positive control for the methylated and unmethylated genes, respectively. PCR products were analyzed on 2% agarose gel and stained with ethidium bromide. Each MSP was repeated at least once to confirm the results.

**Table 1:** Oligonucleotide primers used for methylation-specific PCR.

Primers and sequences (5′ to 3′)	Amplicon (bp)	Annealing temperature (°C)	References
Methylated			
*P16* (F): TTATTAGAGGGTGGGGCGGATCGC	150	58	49
*P16* (R): GACCCCGAACCGCGACCGTAA			
*hMLH1* (F): ACGTAGACGTTTTATTAGGGTCGC	115	58	49
*hMLH1* (R): CCTCATCGTAACTACCCGCG			
*E-cadherin* (F): TGTAGTTACGTATTTATTTTTAGTGGCGTC	112	57.5	50
*E-cadherin* (R): CGA ATA CGA TCG AAT CGA ACC G			
Unmethylated			
*P16* (F): TTATTAGAGGGTGGGGTGGATTGT	151	58	49
*P16* (R): CAACCCCAAACCACAACCATAA			
*hMLH1* (F): TTTTGATGTAGATGTTTTATTAGGGTTGT	124	56	49
*hMLH1* (R): ACCACCTCATCATAACTACCCACA			
*E-cadherin* (F): TGGTTGTAGTTATGTATTTATTTTTAGTGGTGTT	120	57.5	50
*E-cadherin* (R): ACACCAAATACAATCAAATCAAACCAAA			

### Quantitative RT-PCR

A549 cells were transfected with pcDNA™6.2/N-EmGFP-DEST vector and the full-length *A. baumannii* Tnp gene cloned in the expression vector. After 48 h of incubation, total cellular RNA was extracted using the RNeasy kit (Qiagen). The integrity of purified mRNA and concentrations of RNAs were measured by agarose gel electrophoresis and spectrophotometer (Eppendorff), respectively. Reverse transcription was performed in a total volume of 20 µl containing the following: 1 µg of total RNA, 2 µl of 10× buffer, 1 µl of dNTP mixture, 20 µM of oligo-dT primer, and 4 U Molony murine leukemia virus (M-MLV) reverse transcriptase. The reaction mixtures were incubated for 1 h at 42°C and the samples were stored at −20°C. qRT-PCR was carried out using the StepOnePlus Real-Time PCR System (Applied Biosystems) according to the manufacturer's protocol. The specific primer set for *E-cadherin* gene (5′-TAC TAT GAT GAA GAA GGA GG-3′ and 5′-CGG AAC CGC TTC CTT CAT AG-3′) and glyceraldehyde 3-phosphate dehydrogenase gene (GAPDH) (5′-GAG GAG TGG GTG TCG CTG TT-3′ and 5′-GGA CCT GAC CTG CCG TCT AG-3′) were used. qRT-PCR was performed in a total volume of 20 µl with the following components: 10 µl of SYBR Green master-mix (Applied Biosystems), 2 µl of each forward and reverse primers (0.5 µM final concentration), 2 µl of cDNA (100 ng), and 6 µl of dH_2_O. The amplification conditions were: initial denaturation (95°C, 10 min), followed by 40 cycles of denaturation (95°C, 15 s), annealing, and extension (60°C, 1 min). Melting curve analysis was used to confirm amplicon specificity. The normalization and quantification of mRNA expression were performed using the StepOne™ Software version2.2 supplied by the manufacturer.
